# Identification of Hub Genes and Biological Mechanisms Associated with Non-Alcoholic Fatty Liver Disease and Triple-Negative Breast Cancer

**DOI:** 10.3390/life13040998

**Published:** 2023-04-12

**Authors:** Jingjin Zhu, Ningning Min, Wenye Gong, Yizhu Chen, Xiru Li

**Affiliations:** 1 School of Medicine, Nankai University, Tianjin 300071, China; 2120211513@mail.nankai.edu.cn (J.Z.); 2120201347@mail.nankai.edu.cn (N.M.); 2 Department of General Surgery, The First Medical Center of Chinese PLA General Hospital, Beijing 100853, China; gongwenye1998@163.com (W.G.); chenyimizhuzi@163.com (Y.C.); 3 Medical School of Chinese PLA, Beijing 100853, China

**Keywords:** triple-negative breast cancer (TNBC), non-alcoholic fatty liver disease (NAFLD), non-alcoholic steatohepatitis (NASH), bioinformatics analysis, hub genes, prognostic value

## Abstract

The relationship between non-alcoholic fatty liver disease (NAFLD) and triple-negative breast cancer (TNBC) has been widely recognized, but the underlying mechanisms are still unknown. The objective of this study was to identify the hub genes associated with NAFLD and TNBC, and to explore the potential co-pathogenesis and prognostic linkage of these two diseases. We used GEO, TCGA, STRING, ssGSEA, and Rstudio to investigate the common differentially expressed genes (DEGs), conduct functional and signaling pathway enrichment analyses, and determine prognostic value between TNBC and NAFLD. GO and KEGG enrichment analyses of the common DEGs showed that they were enriched in leukocyte aggregation, migration and adhesion, apoptosis regulation, and the PPAR signaling pathway. Fourteen candidate hub genes most likely to mediate NAFLD and TNBC occurrence were identified and validation results in a new cohort showed that ITGB2, RAC2, ITGAM, and CYBA were upregulated in both diseases. A univariate Cox analysis suggested that high expression levels of ITGB2, RAC2, ITGAM, and CXCL10 were associated with a good prognosis in TNBC. Immune infiltration analysis of TNBC samples showed that NCF2, ICAM1, and CXCL10 were significantly associated with activated CD8 T cells and activated CD4 T cells. NCF2, CXCL10, and CYBB were correlated with regulatory T cells and myeloid-derived suppressor cells. This study demonstrated that the redox reactions regulated by the NADPH oxidase (NOX) subunit genes and the transport and activation of immune cells regulated by integrins may play a central role in the co-occurrence trend of NAFLD and TNBC. Additionally, ITGB2, RAC2, and ITGAM were upregulated in both diseases and were prognostic protective factors of TNBC; they may be potential therapeutic targets for treatment of TNBC patients with NAFLD, but further experimental studies are still needed.

## 1. Introduction

Breast cancer is the most commonly diagnosed cancer and the leading cause of cancer death among women [1]. Triple-negative breast cancer (TNBC), defined by estrogen receptor (ER)-negative, progesterone receptor (PR)-negative, and human epidermal growth factor receptor-2 (HER2)-negative histological presentation, accounts for approximately 20% of all breast cancer cases [2,3]. In contrast to other breast cancer types, TNBC has a more aggressive expression profile (high p53 and Ki67 and low Bcl-2 expression), large tumor size, and high histological grade, and is associated with an increased risk of early relapse and poor prognosis [4]. Many potential risk factors for TNBC have been reported, including non-modifiable factors such as age, sex, race, genetic mutations, breast tissue density, and family history of breast disease, and modifiable factors such as diet, lifestyle, obesity, and hormone replacement therapy [5,6,7]. In particular, the increasing proportion of obesity worldwide has led to a sharp rise in patients with metabolic syndrome and an increased risk of certain malignancies [8,9]. It has been shown that metabolic syndrome is positively associated with breast cancer and significantly associated with TNBC [10].

Non-alcoholic fatty liver disease (NAFLD) is a common chronic liver disease intimately related to metabolic syndrome and abdominal obesity [11,12,13]. It is becoming increasingly evident that NAFLD is not only linked to an increased risk of liver-related mortality or morbidity, but also associated with extrahepatic complications such as cardiovascular disease, chronic kidney disease, pulmonary insufficiency, and extrahepatic malignancies [14,15,16]. A retrospective study by Nseir et al. [17] indicated that NAFLD is associated with breast cancer independent of known risk factors.

In addition, breast cancer patients often develop non-alcoholic fatty liver disease during the course of disease. Bilici et al. [18] reported a prevalence of NAFLD as high as 63% and 72% in newly diagnosed and systematically treated breast cancer patients, respectively. It has also been reported that long-term selective estrogen receptor modulator (SERM) administration may increase the risk of NAFLD development, with fatty liver reported in 48.5% of tamoxifen-treated and 50.2% of toremifene-treated breast cancer patients at 60 months [19]. It is of particular note that patients who present with NAFLD have been reported to have longer disease-free survival (DFS) [20,21], but the underlying mechanisms associated with improved clinical outcomes have not been thoroughly investigated.

Recently, advances in sequencing technology and bioinformatics have made it possible to explore the pathogenesis of diseases and the interactions between different diseases at the gene level, which is expected to shed new light on the pathogenesis, diagnosis, and treatment of diseases [22,23,24]. Here, we investigated the common differentially expressed genes (DEGs) of NAFLD and TNBC from public RNA-sequencing databases and identified 14 candidate hub genes most likely to mediate NAFLD and TNBC occurrence. Next, the biological functional pathways of the hub genes were estimated to explore the underlying mechanisms of both diseases. Finally, validation and prognostic analysis were performed in a new cohort of TNBC patients.

## 2. Materials and Methods

### 2.1. Study Design and Data Collection

Three microarray datasets, GSE63067 and GSE48452 of NAFLD, and GSE38959 of TNBC, were collected from the GEO (http://www.ncbi.nlm.nih.gov/geo/, accessed on 1 November 2022) database. The nature of the three microarray datasets from the GEO database is summarized in Table 1. Non-alcoholic steatohepatitis (NASH) is a stage of NAFLD that is usually associated with a worse prognosis [25] and has a higher prevalence and more advanced stage of neoplasms compared to steatosis [26]; thus, the NASH samples were selected as the representatives for analysis in this study. The GSE63067 dataset included the gene expression profiles of 18 samples, of which 9 were NASH patients and 7 were controls, and the GSE38959 dataset included 47 samples, of which 30 were TNBC patients and 13 were controls. The GSE48452 dataset consisted of human liver biopsy samples taken at different phases from control to NASH; 14 controls and 18 NASH samples were used in this study.

RNAseq profiling in the form of fragments per kilobase million (FPKM) and clinicopathological breast cancer data were obtained from the TCGA (https://portal.gdc.cancer.gov, accessed on 1 November 2022) database (TCGA-BRCA cohort). TNBC samples were selected according to the status of ER, PR, and HER2 by referring to the method of Craven et al. [27]. One sample with an unknown ID was excluded, and samples from 132 patients were finally selected for analysis.

### 2.2. Differentially Expressed Gene (DEG) Selection

DEGs were extracted and analyzed separately using the R package “limma”. The fold changes (FCs) were calculated for individual gene expression levels. Genes meeting specific cut-off criteria of *p*-value < 0.05 and |logFC| > with [mean|logFC|) + 2 × sd(|logFC|)] were defined as DEGs. The overlapping DEGs between NAFLD and TNBC were delineated using the R package “ggVennDiagram”. These common DEGs with consistent upregulation or downregulation trends were retained for subsequent analysis.

### 2.3. Functional Classification and Pathway Enrichment of DEGs

The above overlapping DEGs were submitted to Gene Ontology (GO) functional enrichment analysis, which consisted of biological process (BP), cellular component (CC), and molecular function (MF) analyses, and to Kyoto Encyclopedia of Genes and Genomes (KEGG) signaling pathway enrichment analysis using the R package “cluster Profiler”. The enriched GO terms and KEGG pathways with an adjusted *p*-value < 0.1 were selected.

### 2.4. Protein–Protein Interaction (PPI) Establishment and Hub Gene Identification

To further explore the interactions among the common genes obtained as described above, the Search Tool for the Retrieval of Interacting Genes (STRING) (http://string-db.org/, accessed on 12 November 2022) was used for PPI network construction. Subsequently, Cytoscape software was used to visualize the PPI network. The Cytoscape plug-in Minimal Common Oncology Data Elements (MCODE, http://apps.cytoscape.org/apps/mcode, accessed on 12 November 2022) was used to screen out key protein expression molecules and multiple topological analysis algorithms in the cytoHubba plug-in (http://hub.iis.sinica.edu.tw/cytohubba/, accessed on 12 November 2022), such as MCC, MNC, Degree, and EPC, were used to screen the hub genes in the PPI network.

### 2.5. Hub Gene Expression Validation and Prognostic Analysis

The expression levels of the identified hub genes were validated in 132 TNBC samples and 113 controls from the TCGA cohort, and 18 NASH samples and 14 controls from the GEO database. The Wilcoxon test was used to compare the data between the two groups, and a two-sided *p*-value < 0.05 was considered significant. According to the median expression level for each gene, the TNBC samples were divided into high- or low-expression groups. Survival analysis was performed using univariate and multivariate Cox regression hazard analysis, providing hazard ratios (HRs) and 95% confidence intervals (CIs), and survival curves were derived using Kaplan–Meier (KM) survival analysis with log-rank tests for comparison.

### 2.6. Immune Infiltration Analysis

The ssGSEA (single-sample gene set enrichment analysis) algorithm is a rank-based method that defines a score representing the degree of absolute enrichment of a particular gene set in each sample [28,29]. The ssGSEA score utilized immune-cell-marker-associated gene sets (http://cis.hku.hk/TISIDB/data/download/CellReports.txt, accessed on 20 November 2022) to quantify the infiltration of immune cells in TNBC tissue and determine the level of immune infiltration in each sample. Pearson’s correlation analysis was used to reveal the relationships between hub genes and immune cells.

## 3. Results

### 3.1. DEG Identification in NASH and TNBC

**Figure 1:**
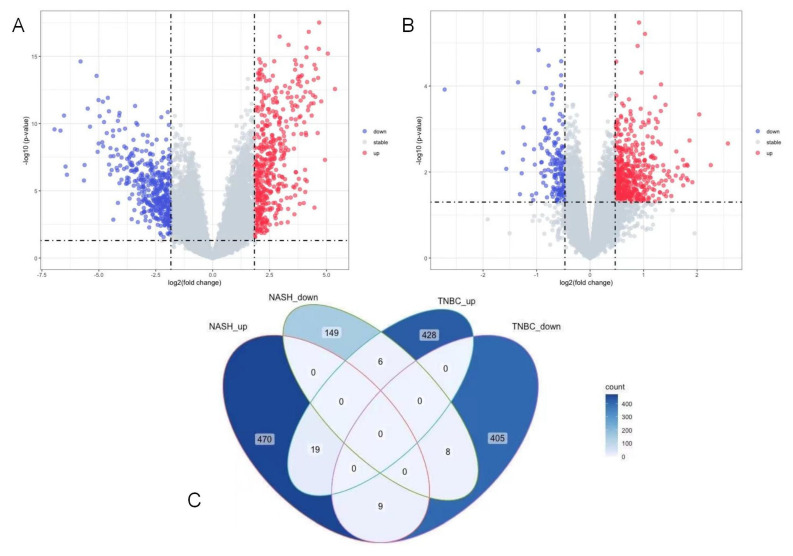
Characterization of the DEGs in NASH and TNBC. (**A**) Volcano map of DEGs between NASH samples and normal samples in GSE63067; (**B**) volcano map of DEGs between TNBC samples and normal samples in GSE38959; (**C**) Venn diagram of the common DEGS between the two upregulation and two downregulation modules in NAFLD and TNBC. DEGs, differentially expressed genes; NASH, non-alcoholic steatohepatitis; TNBC, triple-negative breast cancer.

In the NASH and control groups in the GSE63067 dataset, there were 498 up-DEGs and 163 down-DEGs screened with a logFC threshold of 0.472 (Figure 1A). In the TNBC and control groups in the GSE38959 dataset, there were 453 up-DEGs and 422 down-DEGs screened with a logFC threshold of 1.834 (Figure 1B). A Venn diagram was used to determine the intersection and 42 common DEGs were identified (Figure 1C). After excluding genes with opposite expression trends, 27 DEGs with the same expression trends were found, including 19 common upregulated genes and 8 common downregulated genes (Table S1).

### 3.2. GO and KEGG Enrichment Pathway Analysis of DEGs

To better understand the biological functions of the identified DEGs, GO and KEGG pathway enrichment analyses were performed. After screening with the threshold of adjusted *p* < 0.1, significantly enriched GO terms and KEGG terms were selected.

**Figure 2:**
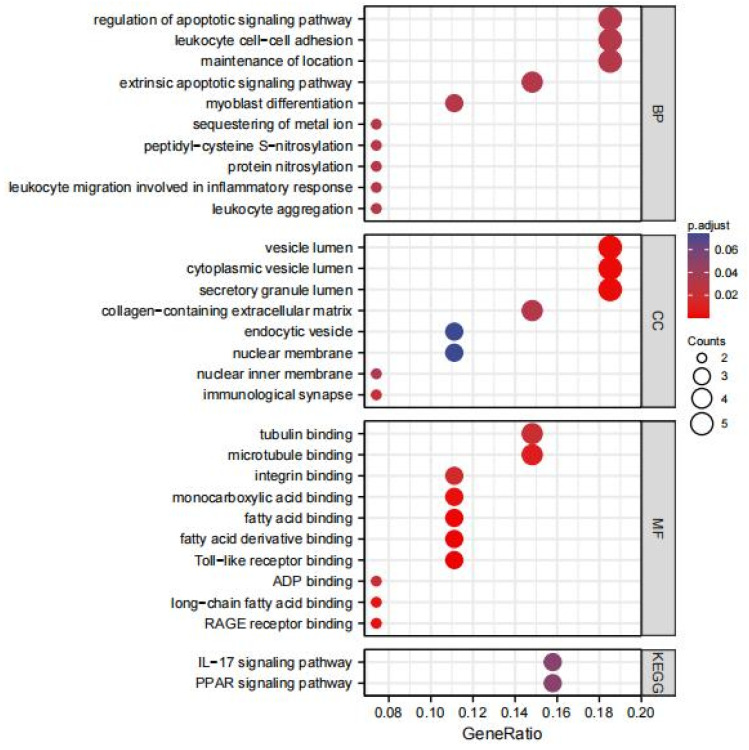
GO and KEGG analysis results of the common DEGs. GO, Gene Ontology; BP, biological process; CC, cellular component; MF, molecular function; KEGG, Kyoto Encyclopedia of Genes and Genomes.

As shown in Figure 2, in the BP category, DEGs were mainly enriched in maintenance of location, leukocyte cell–cell adhesion, leukocyte aggregation, leukocyte migration involved in inflammatory response, protein nitrosylation, peptidyl-cysteine S-nitrosylation, and regulation of apoptotic signaling pathway. In the CC category, DEGs were principally associated with secretory granule lumen, cytoplasmic vesicle lumen, vesicle lumen, immunological synapse, collagen-containing extracellular matrix, and nuclear inner membrane. The analysis of the MF category indicated that DEGs were enriched in toll-like receptor binding, fatty acid derivative binding, fatty acid binding, monocarboxylic acid binding, microtubule binding, integrin binding, and tubulin binding. Furthermore, two KEGG pathways with significant enrichment were the peroxisome proliferator-activated receptor (PPAR) signaling pathway and the IL-17 signaling pathway.

### 3.3. PPI Network Construction and Hub Gene Identification

**Figure 3:**
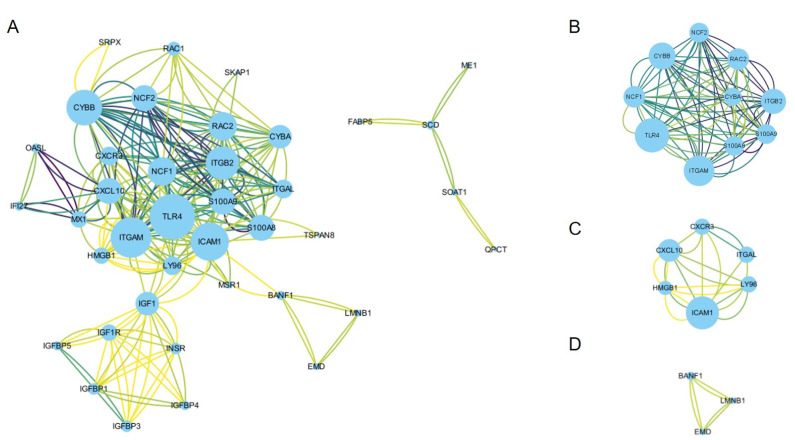
Construction of the PPI network and module analysis. (**A**) The visualization results of the PPI network of the common DEGs obtained from Cytoscape software v3.9.1; (**B**–**D**) three crucial clustering modules extracted by MCODE. PPI, protein–protein interaction; DEG, differentially expressed genes; STRING, Search Tool for the Retrieval of Interacting Genes; MCODE, Minimal Common Oncology Data Elements.

To determine the interactions among the DEGs and identify hub genes, the PPI network of the DEGs was generated using STRING. With the aim of preventing important hub genes being missed, we modified the PPI settings to have a minimum required interaction score of medium confidence (0.400) and a maximum number of interactions of no more than 20 interactors to increase the maximum number of interactions and the number of proteins directly related to the input proteins. Then, a PPI with 47 nodes and 122 edges, with a PPI enrichment *p* value < 1.0 × 10^−16^, was obtained and imported into Cytoscape software v3.9.1 for visualization (Figure 3A, Table S2).

The MCODE plug-in was used to conduct module analysis to detect crucial clustering modules. Three modules were retrieved from the PPI network. The criteria were set as follows: Degree Cutoff = 2, Node Score Cutoff = 0.2, K-Core = 2, and Max. Depth = 100. Module 1 included 10 nodes and 84 edges with a cluster score (density times the number of members) of 9.333. Module 2 and module 3 had 6 nodes and 20 edges and 3 nodes and 6 edges, respectively, and the scores were 4.000 and 3.000, respectively (Figure 3B–D).

The CytoHubba plug-in was used to identify hub genes. Based on the MCC, MNC, Degree, and EPC algorithms, the top 15 important hub genes in the PPI networks were predicted. The intersection of these 15 genes from the four algorithms revealed 14 candidate hub genes: TLR4, CYBB, NCF1, NCF2, S100A8, S100A9, ITGB2, RAC2, ITGAM, CYBA, ICAM1, CXCL10, CXCR3, and ITGAL.

**Figure 4:**
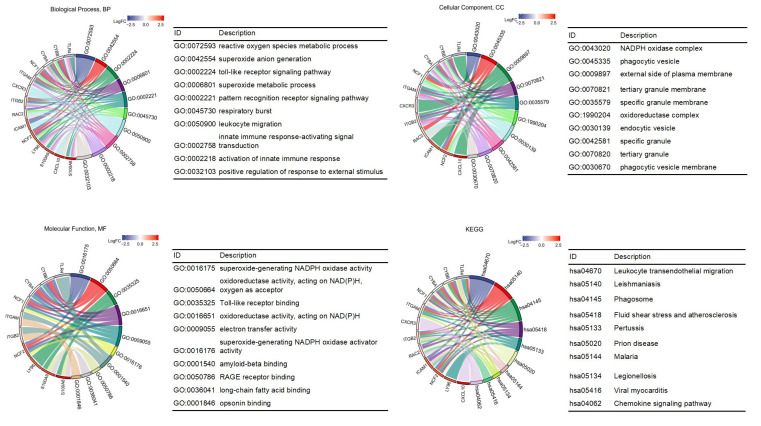
Functional enrichment analysis results of the hub genes. NADPH, nicotinamide adenine dinucleotide phosphate; RAGE, receptor for advanced glycation endproducts.

Combined with the logFC values of the hub genes in the GSE38959 dataset, the GO and KEGG enrichment pathways were analyzed. The top 10 GO terms and KEGG pathways are shown in Figure 4. In the BP category, nine hub genes including CYBB, NCF1, NCF2, ITGB2, RAC2, ITGAM, CYBA, ICAM1, and TLR4 were enriched in reactive oxygen species metabolic process. Furthermore, S100A8, S100A9, ITGB2, RAC2, ITGAM, ICAM1, CXCL10, and CXCR3 were enriched in leukocyte migration. Enriched CC and MF were related to redox reactions like NADPH oxidase complex, superoxide-generating NADPH oxidase activity, and oxidoreductase activity. KEGG enrichment analyses showed that leukocyte transendothelial migration was highly correlated with these genes.

### 3.4. Hub Gene Expression Validation and Prognostic Analysis

**Figure 5:**
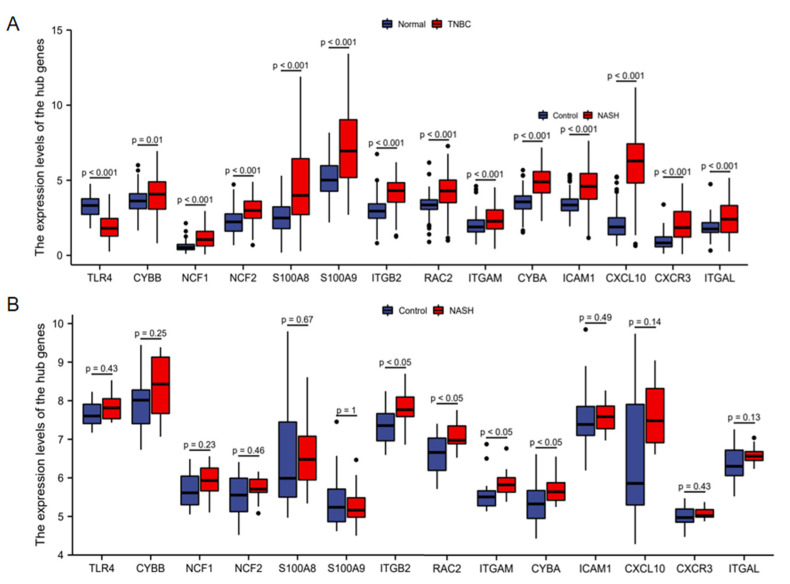
Comparison of hub genes between normal tissues and TNBC samples in TCGA-BRCA cohort (**A**) and control group and NASH samples in the GSE48452 dataset (**B**). TNBC, triple-negative breast cancer; TCGA, The Cancer Genome Atlas; BRCA, breast cancer; NASH, non-alcoholic steatohepatitis; TNBC, triple-negative breast cancer.

Validation was performed in the TCGA-BRCA cohort for TNBC and the GSE48452 dataset for NASH. For TNBC, the differences in the expression levels of all hub genes between normal tissues and TNBC samples were statistically significant (Figure 5A). Compared with normal tissues, 13 hub genes were upregulated, including CYBB, NCF1, NCF2, S100A8, S100A9, ITGB2, RAC2, ITGAM, CYBA, ICAM1, CXCL10, CXCR3, and ITGAL, and TLR4 was downregulated. For NASH, the expressions of hub genes ITGB2, RAC2, ITGAM, and CYBA were upregulated, and the changes in other genes’ expressions were not statistically significant (Figure 5B).

**Figure 6:**
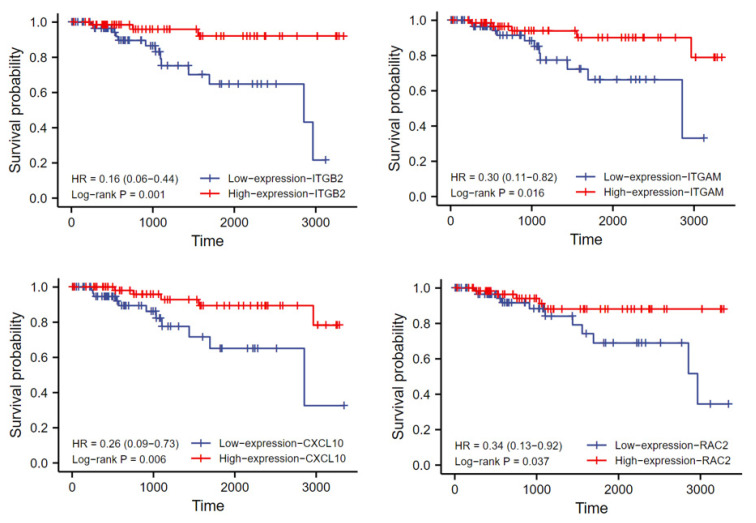
Kaplan–Meier survival curves of association between the expression levels of hub genes and the OS of TNBC patients (group cutoff = median). OS, overall survival; TNBC, triple-negative breast cancer.

To evaluate the clinical relevance of hub gene expression, the TNBC samples were divided into a high-expression group and a low-expression group according to the median expression level of each gene for prognostic analysis. Univariate Cox analysis suggested that high expression of ITGB2, RAC2, ITGAM, and CXCL10 was associated with better overall survival (OS) (Table 2). The corresponding KM survival curves are shown in Figure 6. In addition, two prognostic factors associated with worse OS were identified in terms of clinicopathological features, namely black or African American and Asian ethnicity, and N stage. All variables significant upon univariate Cox regression analysis (*p* ≤ 0.05) were subjected to multivariate Cox regression analysis, and it was found that the N stage was an independent risk factor for overall survival and the remaining factors were not significant.

### 3.5. Association between the Hub Genes and Immune Infiltration

**Figure 7:**
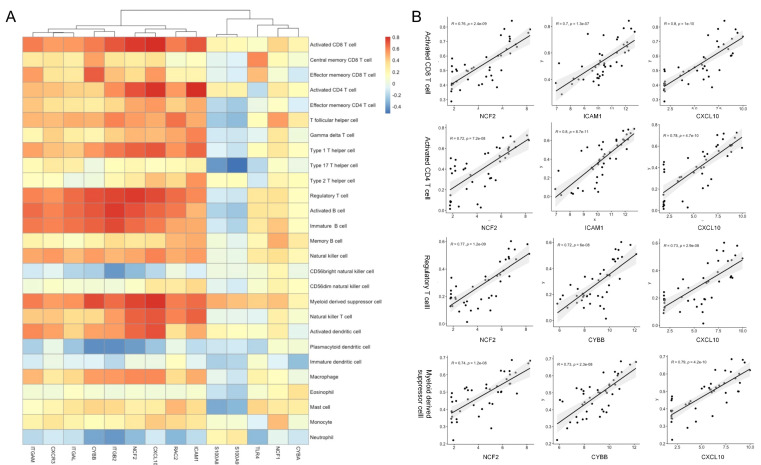
Associations between the hub genes and immune infiltration in the GSE38959 group (**A**) and genes significantly associated with activated CD8 T cells, activated CD4 T cells, regulatory T cells and myeloid-derived suppressor cells (**B**). Red: positive correlation; blue: negative correlation.

Figure 7 shows the relationships between 14 hub genes and 27 immune cells (results for central memory CD4 T cells unavailable) according to the results of ssGSEA analysis. For TNBC samples from the GSE38959 dataset, NCF2, ICAM1, and CXCL10 were significantly associated with activated CD8 T cells (NCF2, r = 0.764, *p* = 2.42 × 10^−9^; ICAM1, r = 0.705, *p* = 1.32 × 10^−7^; CXCL10, r = 0.802, *p* = 1.03 × 10^−10^) and activated CD4 T cells (NCF2, r = 0.715, *p* = 7.16 × 10^−8^; ICAM1, r = 0.804, *p* = 8.70 × 10^−11^; CXCL10, r = 0.785, *p* = 4.72 × 10^−10^). Specifically, NCF2, CXCL10, and CYBB were correlated with regulatory T cells (NCF2, r = 0.773, *p* = 1.22 × 10^−9^; CXCL10, r = 0.730, *p* = 2.86 × 10^−8^; CYBB, r = 0.718, *p* = 6.02 × 10^−8^), and myeloid-derived suppressor cells (MDSCs) (NCF2, r = 0.743, *p* = 1.19 × 10^−8^; CXCL10, r = 0.79, *p* = 4.24 × 10^−10^; CYBB, r = 0.733, *p* = 2.29 × 10^−8^). In addition, ITGB2 was associated with activated B cells (r = 0.766, *p* = 2.22 × 10^−9^) and immature B cells (r = 0.733, *p* = 2.36 × 10^−8^).

## 4. Discussion

In recent years, the relationship between NAFLD and breast cancer has become a research hotspot, and an increasing number of studies have confirmed the correlation. Some studies have reported that breast cancer is a common extrahepatic complication of NAFLD [16,30]. Simultaneously, it also has been suggested that patients with breast cancer, especially those receiving endocrine therapy, present an increased risk of NAFLD [31,32]. Based on these combined results, NAFLD may be related to the occurrence and progression of breast cancer. In addition, it has been proposed that liver metastasis in the diagnosis of fatty liver patients with breast cancer is significantly lower than that of patients with normal liver histology, further revealing the correlation between the two events in clinical practice [33]. However, these studies have been mostly observational, and the mechanism connecting NAFLD and TNBC remains unclear to date. Therefore, exploring the molecular mechanisms to enable early identification and intervention is undoubtedly of great clinical significance.

In this study, we explored common DEGs of NASH and TNBC datasets in public databases through bioinformatics analysis and observed the biological processes and signaling pathways in which they jointly participate. GO and KEGG enrichment analyses of the common DEGs showed enrichment in leukocyte aggregation, migration and adhesion, apoptosis regulation, and the PPAR signaling pathway, suggesting that TNBC in NAFLD patients was likely due to enhanced leukocyte recruitment in the inflammatory response and abnormal apoptosis. Interestingly, the PPAR signaling pathway not only controls the expression of genes encoding proteins of lipid metabolism, but is also involved in anti-cancer responses [34]. One of the mechanisms by which PPARs act to control cancer progression is to affect the NF-κB signaling pathway, or its upstream pathways, such as the Toll-like receptor 4 (TLR4) signaling pathway [35,36]. PPAR γ agonists have been found to induce apoptosis in TNBC cells and inhibit melanoma progression in mice [37,38].

A total of 14 candidate hub genes most likely to mediate NASH and TNBC occurrence were identified, including TLR4, CYBB, NCF1, NCF2, S100A8, S100A9, ITGB2, RAC2, ITGAM, CYBA, ICAM1, CXCL10, CXCR3, and ITGAL.

CYBB, CYBA, NCF1, NCF2, and RAC2 are NADPH oxidase (NOX) subunit genes and are associated with inflammation and fibrosis in multiple organs, such as the liver [39,40], lungs [41], and kidneys [42], as well as with various types of cancer [43]. NOX can produce reactive oxygen species (ROS) that cause changes in cellular redox status, leading to chronic liver injury and fibrosis, which is critical for alcoholic steatohepatitis and NASH [44,45]. The analysis of the TCGA cohort showed that NOX-related genes were expressed more highly in tumor cells than in normal tissues of the same tissue origin, which suggested that the abnormal expression and regulation of NOX may be related to tumorigenesis and the increase of ROS in tumor cells [46], which probably contributes to the increased susceptibility of TNBC patients to NAFLD compared to the healthy population. In addition, RAC2 was strongly associated with OS in patients. RAC2 is a 21 kDa RAS superfamily of GTPases that stabilize the cytoskeleton structure of actin [47,48]. Chen et al. [49] found the high expression of RAC2 can inhibit the proliferation of breast cancer cells.

ITGAM, ITGB2, and ITGAL are involved in the most common integrins expressed on leukocytes, including Mac-1 (αMβ2 or CD11b/CD18) and leukocyte function-related antigen 1 (LFA-1 or αLβ2) [50,51]. Activated integrins play a crucial role in trafficking immune cells into tissues, activating and promoting the proliferation of effector cells, and inducing the formation of immune synapses between cells [52,53]. Clinically, Mac-1 expression is increased in patients with metabolic syndrome [54]. It has also been confirmed that Mac-1 is required for pro-inflammatory gene expression by macrophages in adipose tissue inflammation and is related to recruiting monocytes from bone marrow and inducing them to transform into M1-like macrophages (pro-inflammatory and usually anti-tumor) to express cytotoxic factors to engulf and destroy tumor cells [55,56,57]. Rojas et al. [51] demonstrated that an integrin marker composed of ITGA4, ITGB2, ITGAX, ITGB7, ITGAM, ITGAL, and ITGA8 had the potential to recognize basal-like breast cancers with immune-infiltrating and favorable prognosis. Our results are similar to this finding, with ITGAM and ITGB2 highly expressed in both diseases and associated with a better prognosis in TNBC. Further analysis of immune infiltration showed a positive correlation between integrin genes and activated B cells and immature B cells.

CXCL10 and its homologous receptor CXCR3 are critical in the development of specific features of the NAFLD phenotype, wherein they are mainly involved in the induction of inflammation, regulation of adipogenesis and oxidative stress, and other related processes [58,59]. In the process of tumor progression, studies have shown that CXCL10 has a dual role. It can not only promote tumor progression by increasing cell proliferation and metastasis, but also exert an anti-malignancy function by inhibiting angiogenesis and influencing the tumor microenvironment [60,61,62]. Sun et al. [63] found that CXCL10 expression was significantly upregulated in mice with melanoma and that CXCL10 promoted the proliferation of monocyte-like MDSCs, leading to an immunosuppressive microenvironment. On the other hand, it has also been demonstrated that tumor-cell-derived CXCL9/CXCL10 regulates the recruitment of T cells in various tumors [64,65,66]. Our study suggested that CXCL10 is positively correlated with MDCSs and activated T cells, and TNBC patients with high CXCL10 expression obtained a better prognosis. Therefore, in the context of NAFLD, CXCL10 may play an anti-tumor role in TNBC, but more in-depth experimental research is still needed.

Although many studies have linked metabolic syndrome to the development of cancer and poor prognosis, it may be a symptom of a general metabolic disorder. Our study explored the relationship between NAFLD and TNBC at the genetic level for the first time, and found that the hub genes ITGB2, RAC2, and ITGAM were upregulated in both diseases and were prognostic protective factors in TNBC. This is inconsistent with our understanding of risk factors such as obesity, a high-fat diet, and NAFLD that promote the occurrence and progression of breast cancer. Therefore, further experimental studies will be of great significance and are expected to find new targets for diagnosis, prognostic assessment, and treatment of TNBC.

The study had several limitations. First, although the role of these genes has been elucidated in multiple studies, the key pathways and hub genes identified have not been validated in experiments. Second, due to the lack of a dataset, the validation of the hub gene was performed in patients with only NAFLD or TNBC, but not in patients with NAFLD combined with TNBC. Third, the relationship between the hub genes and the prognosis of TNBC patients needs to be confirmed by prospective clinical studies.

## 5. Conclusions

In conclusion, this study explored the hub genes of NAFLD and TNBC and illustrated the possible mechanisms for the co-occurrence trend of these two diseases. Redox reactions regulated by the NOX subunit genes and the transport and activation of immune cells regulated by integrins may play a central role in the development of NAFLD and TNBC. Additionally, the expressions of ITGB2, RAC2, ITGAM, and CXCL10 were significantly correlated with a good prognosis in TNBC and may be potential therapeutic targets for the development of gene therapies for TNBC patients with NAFLD.

## Figures and Tables

**Table 1:** The nature of the three microarray datasets from the GEO database.

Series	Country	Status	Platforms	Type of Samples	Numbers
GSE63067	Sweden	Public on 7 November 2014	GPL570	non-alcoholic steatohepatitis	9
				steatosis	2
				healthy	7
GSE48452	Germany	Public on 8 August 2013	GPL11532	non-alcoholic steatohepatitis	18
				steatosis	14
				healthy obese	27
				control	14
GSE38959	Japan	Public on 21 December 2012	GPL4133	triple-negative breast cancer	30
				normal mammary ductal cells	13
				normal human vital organs including heart, lung, liver, and kidney	4

**Table 2:** The Cox regression analysis results of the hub genes and clinicopathological variables in the TCGA-BRCA group.

Factor	Univariate Cox Regression Analysis		Multivariate Cox Regression Analysis	
	HR (95%CI)	*p*-Value	HR (95%CI)	*p*-Value
TLR4	0.640 (0.237–1.728)	0.378		
CYBB	0.509 (0.183–1.411)	0.194		
NCF1	0.431 (0.155–1.200)	0.107		
NCF2	0.622 (0.228–1.694)	0.352		
S100A8	0.810 (0.295–2.226)	0.683		
S100A9	0.523 (0.181–1.507)	0.230		
ITGB2	0.157 (0.044–0.556)	0.004	0.213 (0.033–1.376)	0.104
RAC2	0.341 (0.118–0.984)	0.047	1.067 (0.282–4.036)	0.924
ITGAM	0.282 (0.094–0.842)	0.023	1.392 (0.318–6.100)	0.661
CYBA	1.750 (0.632–4.847)	0.282		
ICAM1	0.676 (0.250–1.828)	0.440		
CXCL10	0.244 (0.082–0.725)	0.011	0.430 (0.108–1.718)	0.232
CXCR3	0.419 (0.152–1.156)	0.093		
ITGAL	0.413 (0.148–1.151)	0.091		
Age	0.773 (0.249–2.406)	0.657		
Race	2.830 (1.019–7.860)	0.046	2.090 (0.631–6.922)	0.227
T stage				
(T2 vs. T1, T3/T4 vs. T1)	1.717 (0.471–6.255)	0.194		
	4.427 (0.858–22.838)			
N stage				
(N1/N2/N3 vs. N0)	5.641 (1.815–17.534)	0.003	4.681 (1.452–15.089)	0.010

## Data Availability

Publicly available datasets were analyzed in this study. The data can be found here: http://www.ncbi.nlm.nih.gov/geo/ (accessed on 1 November 2022), and https://portal.gdc.cancer.gov (accessed on 1 November 2022).

## Supplementary Materials

The following supporting information can be downloaded at: https://www.mdpi.com/article/10.3390/life13040998/s1; Table S1. The common DEGs; Table S2. The details of the PPI obtained from STRING database analysis.

## References

[1] Sung H., Ferlay J., Siegel R.L., Laversanne M., Soerjomataram I., Jemal A., Bray F. (2021). Global Cancer Statistics 2020: GLOBOCAN Estimates of Incidence and Mortality Worldwide for 36 Cancers in 185 Countries. CA Cancer J. Clin..

[2] Billar J.A., Dueck A.C., Stucky C.C., Gray R.J., Wasif N., Northfelt D.W., McCullough A.E., Pockaj B.A. (2010). Triple-negative breast cancers: Unique clinical presentations and outcomes. Ann. Surg. Oncol..

[3] Yin L., Duan J.J., Bian X.W., Yu S.C. (2020). Triple-negative breast cancer molecular subtyping and treatment progress. Breast Cancer Res..

[4] Nofech-Mozes S., Trudeau M., Kahn H.K., Dent R., Rawlinson E., Sun P., Narod S.A., Hanna W.M. (2009). Patterns of recurrence in the basal and non-basal subtypes of triple-negative breast cancers. Breast Cancer Res. Treat.

[5] Almansour N.M. (2022). Triple-Negative Breast Cancer: A Brief Review About Epidemiology, Risk Factors, Signaling Pathways, Treatment and Role of Artificial Intelligence. Front. Mol. Biosci..

[6] Cornejo-Moreno B.A., Uribe-Escamilla D., Salamanca-Gómez F. (2014). Breast cancer genes: Looking for BRACA’s lost brother. Isr. Med. Assoc. J..

[7] Toro A.L., Costantino N.S., Shriver C.D., Ellsworth D.L., Ellsworth R.E. (2016). Effect of obesity on molecular characteristics of invasive breast tumors: Gene expression analysis in a large cohort of female patients. BMC Obes..

[8] Wang C.H., Lundh M., Fu A., Kriszt R., Huang T.L., Lynes M.D., Leiria L.O., Shamsi F., Darcy J., Greenwood B.P. (2020). CRISPR-engineered human brown-like adipocytes prevent diet-induced obesity and ameliorate metabolic syndrome in mice. Sci. Transl. Med..

[9] Kazak L., Chouchani E.T., Lu G.Z., Jedrychowski M.P., Bare C.J., Mina A.I., Kumari M., Zhang S., Vuckovic I., Laznik-Bogoslavski D. (2017). Genetic Depletion of Adipocyte Creatine Metabolism Inhibits Diet-Induced Thermogenesis and Drives Obesity. Cell Metab..

[10] Akinyemiju T., Oyekunle T., Salako O., Gupta A., Alatise O., Ogun G., Adeniyi A., Deveaux A., Hall A., Ayandipo O. (2022). Metabolic Syndrome and Risk of Breast Cancer by Molecular Subtype: Analysis of the MEND Study. Clin. Breast Cancer.

[11] Chalasani N., Younossi Z., Lavine J.E., Diehl A.M., Brunt E.M., Cusi K., Charlton M., Sanyal A.J. (2012). The diagnosis and management of non-alcoholic fatty liver disease: Practice guideline by the American Gastroenterological Association, American Association for the Study of Liver Diseases, and American College of Gastroenterology. Gastroenterology.

[12] Lomonaco R., Sunny N.E., Bril F., Cusi K. (2013). Nonalcoholic fatty liver disease: Current issues and novel treatment approaches. Drugs.

[13] Younossi Z.M., Koenig A.B., Abdelatif D., Fazel Y., Henry L., Wymer M. (2016). Global epidemiology of nonalcoholic fatty liver disease-Meta-analytic assessment of prevalence, incidence, and outcomes. Hepatology.

[14] Wei Z., Ren Z., Hu S., Gao Y., Sun R., Lv S., Yang G., Yu Z., Kan Q. (2020). Development and validation of a simple risk model to predict major cancers for patients with nonalcoholic fatty liver disease. Cancer Med..

[15] Simon T.G., Roelstraete B., Sharma R., Khalili H., Hagström H., Ludvigsson J.F. (2021). Cancer Risk in Patients With Biopsy-ConfirMed. Nonalcoholic Fatty Liver Disease: A Population-Based Cohort Study. Hepatology.

[16] Hong C., Yan Y., Su L., Chen D., Zhang C. (2022). Development of a risk-stratification scoring system for predicting risk of breast cancer based on non-alcoholic fatty liver disease, non-alcoholic fatty pancreas disease, and uric acid. Open Med. (Wars).

[17] Nseir W., Abu-Rahmeh Z., Tsipis A., Mograbi J., Mahamid M. (2017). Relationship between Non-Alcoholic Fatty Liver Disease and Breast Cancer. Isr. Med. Assoc. J..

[18] Bilici A., Ozguroglu M., Mihmanli I., Turna H., Adaletli I. (2007). A case-control study of non-alcoholic fatty liver disease in breast cancer. Med. Oncol..

[19] Yang Y.J., Kim K.M., An J.H., Lee D.B., Shim J.H., Lim Y.S., Lee H.C., Lee Y.S., Ahn J.H., Jung K.H. (2016). Clinical significance of fatty liver disease induced by tamoxifen and toremifene in breast cancer patients. Breast.

[20] Taroeno-Hariadi K.W., Putra Y.R., Choridah L., Widodo I., Hardianti M.S., Aryandono T. (2021). Fatty Liver in Hormone Receptor-Positive Breast Cancer and Its Impact on Patient’s Survival. J. Breast Cancer.

[21] Zheng Q., Xu F., Nie M., Xia W., Qin T., Qin G., An X., Xue C., Peng R., Yuan Z. (2015). Selective Estrogen Receptor Modulator-Associated Nonalcoholic Fatty Liver Disease Improved Survival in Patients With Breast Cancer: A Retrospective Cohort Analysis. Medicine (Baltim.).

[22] Li H., Yu L., Zhang X., Shang J., Duan X. (2022). Exploring the molecular mechanisms and shared gene signatuRes. between rheumatoid arthritis and diffuse large B cell lymphoma. Front. Immunol..

[23] Hu Y., Zeng N., Ge Y., Wang D., Qin X., Zhang W., Jiang F., Liu Y. (2022). Identification of the Shared Gene SignatuRes. and Biological Mechanism in Type 2 Diabetes and Pancreatic Cancer. Front. Endocrinol. (Lausanne).

[24] Chen Y., Ma L., Ge Z., Pan Y., Xie L. (2022). Key Genes Associated With Non-Alcoholic Fatty Liver Disease and Polycystic Ovary Syndrome. Front. Mol. Biosci..

[25] Cotter T.G., Rinella M. (2020). Nonalcoholic Fatty Liver Disease 2020: The State of the Disease. Gastroenterology.

[26] Wong V.W., Wong G.L., Tsang S.W., Fan T., Chu W.C., Woo J., Chan A.W., Choi P.C., Chim A.M., Lau J.Y. (2011). High prevalence of colorectal neoplasm in patients with non-alcoholic steatohepatitis. Gut.

[27] Craven K.E., Gökmen-Polar Y., Badve S.S. (2021). CIBERSORT analysis of TCGA and METABRIC identifies subgroups with better outcomes in triple negative breast cancer. Sci. Rep..

[28] Finotello F., Trajanoski Z. (2018). Quantifying tumor-infiltrating immune cells from transcriptomics data. Cancer Immunol. Immunother..

[29] Bindea G., Mlecnik B., Tosolini M., Kirilovsky A., Waldner M., Obenauf A.C., Angell H., Fredriksen T., Lafontaine L., Berger A. (2013). Spatiotemporal dynamics of intratumoral immune cells reveal the immune landscape in human cancer. Immunity.

[30] Sanna C., Rosso C., Marietti M., Bugianesi E. (2016). Non-Alcoholic Fatty Liver Disease and Extra-Hepatic Cancers. Int. J. Mol. Sci..

[31] Pan H.J., Chang H.T., Lee C.H. (2016). Association between tamoxifen treatment and the development of different stages of nonalcoholic fatty liver disease among breast cancer patients. J. Formos. Med. Assoc..

[32] Yan M., Wang J., Xuan Q., Dong T., He J., Zhang Q. (2017). The Relationship Between Tamoxifen-associated Nonalcoholic Fatty Liver Disease and the Prognosis of Patients With Early-stage Breast Cancer. Clin. Breast Cancer.

[33] Wu W., Chen J., Ye W., Li X., Zhang J. (2017). Fatty liver decreases the risk of liver metastasis in patients with breast cancer: A two-center cohort study. Breast Cancer Res. Treat.

[34] Chinetti G., Fruchart J.C., Staels B. (2000). Peroxisome proliferator-activated receptors (PPARs): Nuclear receptors at the crossroads between lipid metabolism and inflammation. Inflamm. Res..

[35] Michalik L., Wahli W. (2008). PPARs Mediate Lipid Signaling in Inflammation and Cancer. PPAR Res..

[36] Zúñiga J., Cancino M., Medina F., Varela P., Vargas R., Tapia G., Videla L.A., Fernández V. (2011). N-3 PUFA supplementation triggers PPAR-α activation and PPAR-α/NF-κB interaction: Anti-inflammatory implications in liver ischemia-reperfusion injury. PLoS ONE.

[37] Dana N., Haghjooy Javanmard S., Vaseghi G. (2020). The effect of fenofibrate, a PPARα activator on toll-like receptor-4 signal transduction in melanoma both in vitro and in vivo. Clin. Transl. Oncol..

[38] Li T., Zhang Q., Zhang J., Yang G., Shao Z., Luo J., Fan M., Ni C., Wu Z., Hu X. (2014). Fenofibrate induces apoptosis of triple-negative breast cancer cells via activation of NF-κB pathway. BMC Cancer.

[39] Crosas-Molist E., Fabregat I. (2015). Role of NADPH oxidases in the redox biology of liver fibrosis. Redox Biol..

[40] García-Ruiz I., Blanes Ruiz N., Rada P., Pardo V., Ruiz L., Blas-García A., Valdecantos M.P., Grau Sanz M., Solís Herruzo J.A., Valverde Á.M. (2019). Protein tyrosine phosphatase 1b deficiency protects against hepatic fibrosis by modulating nadph oxidases. Redox Biol..

[41] Hecker L., Vittal R., Jones T., Jagirdar R., Luckhardt T.R., Horowitz J.C., Pennathur S., Martinez F.J., Thannickal V.J. (2009). NADPH oxidase-4 mediates myofibroblast activation and fibrogenic responses to lung injury. Nat. Med..

[42] Bondi C.D., Manickam N., Lee D.Y., Block K., Gorin Y., Abboud H.E., Barnes J.L. (2010). NAD(P)H oxidase mediates TGF-beta1-induced activation of kidney myofibroblasts. J. Am. Soc. Nephrol..

[43] Panday A., Sahoo M.K., Osorio D., Batra S. (2015). NADPH oxidases: An overview from structure to innate immunity-associated pathologies. Cell Mol. Immunol..

[44] Matuz-Mares D., Vázquez-Meza H., Vilchis-Landeros M.M. (2022). NOX as a Therapeutic Target in Liver Disease. Antioxidants.

[45] Ma Y., Lee G., Heo S.Y., Roh Y.S. (2021). Oxidative Stress Is a Key Modulator in the Development of Nonalcoholic Fatty Liver Disease. Antioxidants.

[46] Bedard K., Krause K.H. (2007). The NOX family of ROS-generating NADPH oxidases: Physiology and pathophysiology. Physiol. Rev..

[47] Arrington M.E., Temple B., Schaefer A., Campbell S.L. (2020). The molecular basis for immune dysregulation by the hyperactivated E62K mutant of the GTPase RAC2. J. Biol. Chem..

[48] Watanabe M., Terasawa M., Miyano K., Yanagihara T., Uruno T., Sanematsu F., Nishikimi A., Côté J.F., Sumimoto H., Fukui Y. (2014). DOCK2 and DOCK5 act additively in neutrophils to regulate chemotaxis, superoxide production, and extracellular trap formation. J. Immunol..

[49] Chen Q., Jun H., Yang C., Yang F., Xu Y. (2022). The Pyroptosis-Related Risk Genes APOBEC3D, TNFRSF14, and RAC2 Were Used to Evaluate Prognosis and as Tumor Suppressor Genes in Breast Cancer. J. Oncol..

[50] Abram C.L., Lowell C.A. (2009). The ins and outs of leukocyte integrin signaling. Annu. Rev. Immunol..

[51] Rojas K., Baliu-Piqué M., Manzano A., Saiz-Ladera C., García-Barberán V., Cimas F.J., Pérez-Segura P., Pandiella A., Győrffy B., Ocana A. (2021). In silico transcriptomic mapping of integrins and immune activation in Basal-like and HER2+ breast cancer. Cell Oncol. (Dordr.).

[52] Rose D.M., Liu S., Woodside D.G., Han J., Schlaepfer D.D., Ginsberg M.H. (2003). Paxillin binding to the alpha 4 integrin subunit stimulates LFA-1 (integrin alpha L beta 2)-dependent T cell migration by augmenting the activation of focal adhesion kinase/proline-rich tyrosine kinase-2. J. Immunol..

[53] Harjunpää H., Llort Asens M., Guenther C., Fagerholm S.C. (2019). Cell Adhesion Molecules and Their Roles and Regulation in the Immune and Tumor Microenvironment. Front. Immunol..

[54] Jialal I., Adams-Huet B., Devaraj S. (2016). Monocyte cell adhesion molecule receptors in nascent metabolic syndrome. Clin. BioChem..

[55] Winer S., Winer D.A. (2012). The adaptive immune system as a fundamental regulator of adipose tissue inflammation and insulin resistance. Immunol. Cell Biol..

[56] Osborn O., Olefsky J.M. (2012). The cellular and signaling networks linking the immune system and metabolism in disease. Nat. Med..

[57] Lumeng C.N., Bodzin J.L., Saltiel A.R. (2007). Obesity induces a phenotypic switch in adipose tissue macrophage polarization. J. Clin. Investig..

[58] Xu Z., Zhang X., Lau J., Yu J. (2016). C-X-C motif chemokine 10 in non-alcoholic steatohepatitis: Role as a pro-inflammatory factor and clinical implication. Expert Rev. Mol. Med..

[59] Zhang X., Han J., Man K., Li X., Du J., Chu E.S., Go M.Y., Sung J.J., Yu J. (2016). CXC chemokine receptor 3 promotes steatohepatitis in mice through mediating inflammatory cytokines, macrophages and autophagy. J. Hepatol..

[60] Wu X., Sun A., Yu W., Hong C., Liu Z. (2020). CXCL10 mediates breast cancer tamoxifen resistance and promotes estrogen-dependent and independent proliferation. Mol. Cell Endocrinol..

[61] Zhou H., Wu J., Wang T., Zhang X., Liu D. (2016). CXCL10/CXCR3 axis promotes the invasion of gastric cancer via PI3K/AKT pathway-dependent MMPs production. BioMed. Pharmacother..

[62] Fujita M., Zhu X., Ueda R., Sasaki K., Kohanbash G., Kastenhuber E.R., McDonald H.A., Gibson G.A., Watkins S.C., Muthuswamy R. (2009). Effective immunotherapy against murine gliomas using type 1 polarizing dendritic cells—significant roles of CXCL10. Cancer Res..

[63] Sun Y., Mo Y., Jiang S., Shang C., Feng Y., Zeng X. (2022). CXC chemokine ligand-10 promotes the accumulation of monocyte-like myeloid-derived suppressor cells by activating p38 MAPK signaling under tumor conditions. Cancer Sci..

[64] Mowat C., Mosley S.R., Namdar A., Schiller D., Baker K. (2021). Anti-tumor immunity in mismatch repair-deficient colorectal cancers requiRes. type I IFN-driven CCL5 and CXCL10. J. Exp. Med..

[65] Reschke R., Gajewski T.F. (2022). CXCL9 and CXCL10 bring the heat to tumors. Sci. Immunol..

[66] Hoch T., Schulz D., Eling N., Gómez J.M., Levesque M.P., Bodenmiller B. (2022). Multiplexed imaging mass cytometry of the chemokine milieus in melanoma characterizes featuRes. of the response to immunotherapy. Sci. Immunol..

