# Supplementary material for: Identification of Hub Genes and Biological Mechanisms Associated with Non-Alcoholic Fatty Liver Disease and Triple-Negative Breast Cancer
Source: Life (Basel). 2023 Apr 12;13(4):998. doi: 10.3390/life13040998 (PMC10146727; doi:10.3390/life13040998)
Supplement: Supplementary file 1 [file life-13-00998-s001.zip › life-2226576-supplementary.pdf]

Supplementary Material

Table S1. The common DEGs.

No.	Name	Trend
1	ARNTL2	up-regulated
2	IFI27	up-regulated
3	FABP5	up-regulated
4	SCD	up-regulated
5	LMNB1	up-regulated
6	ME1	up-regulated
7	ICAM1	up-regulated
8	MSR1	up-regulated
9	TAP1	up-regulated
10	SOAT1	up-regulated
11	S100A8	up-regulated
12	S100A9	up-regulated
13	PIM1	up-regulated
14	PCOLCE2	up-regulated
15	QPCT	up-regulated
16	LY96	up-regulated
17	CXCL10	up-regulated
18	MX1	up-regulated
19	NCF2	up-regulated
20	TBX3	down-regulated
21	TSPAN8	down-regulated
22	SKAP1	down-regulated
23	FMO2	down-regulated
24	SAMD5	down-regulated

25	IGF1	down-regulated
26	FAM47E	down-regulated
27	SRPX	down-regulated

Table S2. The details of PPI obtained from STRING database analysis.

#node1	node2	node1_string_id	node2_string_id	neighborhood_on_chromosome	gene_fusion	phylogenetic_cooccurrence	homology	coexpression	experimentally_determined_interaction	database_annotated	automated_textmining	combined_score
BANF1	LMNB1	9606.ENSP00000310275	9606.ENSP00000261366	0	0	0	0	0.063	0.378	0.9	0.986	0.999
BANF1	ICAM1	9606.ENSP00000310275	9606.ENSP00000264832	0	0	0	0	0	0	0	0.424	0.424
BANF1	EMD	9606.ENSP00000310275	9606.ENSP00000358857	0	0	0	0	0.063	0.975	0.6	0.989	0.999
CXCL10	OASL	9606.ENSP00000305651	9606.ENSP00000257570	0	0	0	0	0.639	0	0	0.522	0.82
CXCL10	ICAM1	9606.ENSP00000305651	9606.ENSP00000264832	0	0	0	0	0.145	0	0	0.708	0.739
CXCL10	LY96	9606.ENSP00000305651	9606.ENSP00000284818	0	0	0	0	0.11	0	0	0.459	0.497
CXCL10	CYBB	9606.ENSP00000305651	9606.ENSP00000367851	0	0	0	0	0.157	0	0	0.324	0.406
CXCL10	HMGB1	9606.ENSP00000305651	9606.ENSP00000345347	0	0	0	0	0	0	0	0.468	0.468
CXCL10	IFI27	9606.ENSP00000305651	9606.ENSP00000483430	0	0	0	0	0.216	0	0	0.459	0.558
CXCL10	ITGAM	9606.ENSP00000305651	9606.ENSP00000441691	0	0	0	0	0.126	0	0	0.672	0.701
CXCL10	MX1	9606.ENSP00000305651	9606.ENSP00000381601	0	0	0	0	0.416	0	0	0.703	0.819
CXCL10	TLR4	9606.ENSP00000305651	9606.ENSP00000363089	0	0	0	0	0.078	0	0	0.959	0.961
CXCL10	CXCR3	9606.ENSP00000305651	9606.ENSP00000362795	0	0	0	0	0.09	0.678	0.9	0.989	0.999
CXCR3	ICAM1	9606.ENSP00000362795	9606.ENSP00000264832	0	0	0	0	0.062	0	0	0.579	0.588
CXCR3	CXCL10	9606.ENSP00000362795	9606.ENSP00000305651	0	0	0	0	0.09	0.678	0.9	0.989	0.999
CXCR3	ITGAL	9606.ENSP00000362795	9606.ENSP00000349252	0	0	0	0	0.209	0	0	0.556	0.633
CXCR3	MX1	9606.ENSP00000362795	9606.ENSP00000381601	0	0	0	0	0.08	0.056	0	0.395	0.429
CXCR3	ITGB2	9606.ENSP00000362795	9606.ENSP00000380948	0	0	0	0	0.213	0	0	0.323	0.444
CXCR3	TLR4	9606.ENSP00000362795	9606.ENSP00000363089	0	0	0	0	0.062	0	0	0.469	0.48
CXCR3	ITGAM	9606.ENSP00000362795	9606.ENSP00000441691	0	0	0	0	0.148	0	0	0.671	0.708

CYBA	RAC2	9606.ENSP00000261623	9606.ENSP00000249071	0	0	0	0	0.227	0	0.8	0.959	0.993
CYBA	TLR4	9606.ENSP00000261623	9606.ENSP00000363089	0	0	0	0	0.062	0	0	0.392	0.405
CYBA	ICAM1	9606.ENSP00000261623	9606.ENSP00000264832	0	0	0	0	0.101	0	0	0.445	0.48
CYBA	S100A8	9606.ENSP00000261623	9606.ENSP00000357722	0	0	0	0	0.087	0	0.6	0.042	0.619
CYBA	S100A9	9606.ENSP00000261623	9606.ENSP00000357727	0	0	0	0	0.087	0	0.6	0.072	0.632
CYBA	RAC1	9606.ENSP00000261623	9606.ENSP00000348461	0	0	0	0	0.064	0.213	0.9	0.409	0.95
CYBA	CYBB	9606.ENSP00000261623	9606.ENSP00000367851	0	0	0	0	0.143	0.302	0.9	0.993	0.999
CYBA	NCF2	9606.ENSP00000261623	9606.ENSP00000356505	0	0	0	0	0.156	0.462	0.9	0.993	0.999
CYBA	NCF1	9606.ENSP00000261623	9606.ENSP00000289473	0	0	0	0	0.155	0.748	0.9	0.993	0.999
CYBB	RAC2	9606.ENSP00000367851	9606.ENSP00000249071	0	0	0	0	0.259	0.237	0.8	0.988	0.998
CYBB	CYBA	9606.ENSP00000367851	9606.ENSP00000261623	0	0	0	0	0.143	0.302	0.9	0.993	0.999
CYBB	ICAM1	9606.ENSP00000367851	9606.ENSP00000264832	0	0	0	0	0.088	0	0	0.572	0.593
CYBB	NCF1	9606.ENSP00000367851	9606.ENSP00000289473	0	0	0	0	0.303	0.213	0.9	0.992	0.999
CYBB	CXCL10	9606.ENSP00000367851	9606.ENSP00000305651	0	0	0	0	0.157	0	0	0.324	0.406
CYBB	RAC1	9606.ENSP00000367851	9606.ENSP00000348461	0	0	0	0	0.093	0.242	0.9	0.62	0.97
CYBB	ITGAL	9606.ENSP00000367851	9606.ENSP00000349252	0	0	0	0	0.458	0	0	0.17	0.531
CYBB	NCF2	9606.ENSP00000367851	9606.ENSP00000356505	0	0	0	0	0.382	0.462	0.9	0.992	0.999
CYBB	S100A8	9606.ENSP00000367851	9606.ENSP00000357722	0	0	0	0	0.347	0	0.6	0.15	0.758
CYBB	S100A9	9606.ENSP00000367851	9606.ENSP00000357727	0	0	0	0	0.351	0	0.6	0.154	0.761
CYBB	TLR4	9606.ENSP00000367851	9606.ENSP00000363089	0	0	0	0	0.296	0.059	0	0.692	0.778
CYBB	SRPX	9606.ENSP00000367851	9606.ENSP00000367794	0	0	0	0	0	0	0	0.536	0.536
CYBB	ITGB2	9606.ENSP00000367851	9606.ENSP00000380948	0	0	0	0	0.568	0	0	0.323	0.695
CYBB	ITGAM	9606.ENSP00000367851	9606.ENSP00000441691	0	0	0	0	0.56	0	0	0.587	0.811
EMD	LMNB1	9606.ENSP00000358857	9606.ENSP00000261366	0	0	0	0	0.084	0.317	0.9	0.988	0.999
EMD	BANF1	9606.ENSP00000358857	9606.ENSP00000310275	0	0	0	0	0.063	0.975	0.6	0.989	0.999
FABP5	SCD	9606.ENSP00000297258	9606.ENSP00000359380	0	0	0	0	0.062	0	0	0.488	0.499
HMGB1	ICAM1	9606.ENSP00000345347	9606.ENSP00000264832	0	0	0	0	0	0	0	0.594	0.594

HMGB1	LY96	9606.ENSP00000345347	9606.ENSP00000284818	0	0	0	0	0	0.417	0.9	0.978	0.998
HMGB1	CXCL10	9606.ENSP00000345347	9606.ENSP00000305651	0	0	0	0	0	0	0	0.468	0.468
HMGB1	ITGB2	9606.ENSP00000345347	9606.ENSP00000380948	0	0	0	0	0.065	0	0.9	0.445	0.943
HMGB1	ITGAM	9606.ENSP00000345347	9606.ENSP00000441691	0	0	0	0	0	0	0.9	0.641	0.962
HMGB1	TLR4	9606.ENSP00000345347	9606.ENSP00000363089	0	0	0	0	0	0.632	0.9	0.989	0.999
ICAM1	RAC2	9606.ENSP00000264832	9606.ENSP00000249071	0	0	0	0	0.125	0	0	0.549	0.589
ICAM1	CYBA	9606.ENSP00000264832	9606.ENSP00000261623	0	0	0	0	0.101	0	0	0.445	0.48
ICAM1	BANF1	9606.ENSP00000264832	9606.ENSP00000310275	0	0	0	0	0	0	0	0.424	0.424
ICAM1	LY96	9606.ENSP00000264832	9606.ENSP00000284818	0	0	0	0	0.135	0	0	0.391	0.45
ICAM1	TSPAN8	9606.ENSP00000264832	9606.ENSP00000377003	0	0	0	0	0.052	0	0	0.472	0.479
ICAM1	IGF1	9606.ENSP00000264832	9606.ENSP00000302665	0	0	0	0	0	0	0	0.516	0.516
ICAM1	NCF1	9606.ENSP00000264832	9606.ENSP00000289473	0	0	0	0	0.069	0	0	0.541	0.555
ICAM1	CXCR3	9606.ENSP00000264832	9606.ENSP00000362795	0	0	0	0	0.062	0	0	0.579	0.588
ICAM1	CYBB	9606.ENSP00000264832	9606.ENSP00000367851	0	0	0	0	0.088	0	0	0.572	0.593
ICAM1	HMGB1	9606.ENSP00000264832	9606.ENSP00000345347	0	0	0	0	0	0	0	0.594	0.594
ICAM1	CXCL10	9606.ENSP00000264832	9606.ENSP00000305651	0	0	0	0	0.145	0	0	0.708	0.739
ICAM1	TLR4	9606.ENSP00000264832	9606.ENSP00000363089	0	0	0	0	0.083	0	0	0.786	0.796
ICAM1	ITGAL	9606.ENSP00000264832	9606.ENSP00000349252	0	0	0	0	0.091	0.972	0.9	0.989	0.999
ICAM1	ITGAM	9606.ENSP00000264832	9606.ENSP00000441691	0	0	0	0	0.096	0.462	0.9	0.989	0.999
ICAM1	ITGB2	9606.ENSP00000264832	9606.ENSP00000380948	0	0	0	0	0.113	0.835	0.9	0.989	0.999
IFI27	OASL	9606.ENSP00000483430	9606.ENSP00000257570	0	0	0	0	0.117	0	0.9	0.67	0.968
IFI27	CXCL10	9606.ENSP00000483430	9606.ENSP00000305651	0	0	0	0	0.216	0	0	0.459	0.558
IFI27	MX1	9606.ENSP00000483430	9606.ENSP00000381601	0	0	0	0	0.657	0	0.9	0.687	0.988
IGF1	IGFBP5	9606.ENSP00000302665	9606.ENSP00000233813	0	0	0	0	0	0.945	0.72	0.99	0.999
IGF1	ICAM1	9606.ENSP00000302665	9606.ENSP00000264832	0	0	0	0	0	0	0	0.516	0.516
IGF1	IGF1R	9606.ENSP00000302665	9606.ENSP00000268035	0	0	0	0	0	0.973	0.9	0.992	0.999
IGF1	IGFBP4	9606.ENSP00000302665	9606.ENSP00000269593	0	0	0	0	0.085	0.973	0.6	0.989	0.999

IGF1	IGFBP1	9606.ENSP00000302665	9606.ENSP00000275525	0	0	0	0	0.062	0.959	0.6	0.991	0.999
IGF1	TLR4	9606.ENSP00000302665	9606.ENSP00000363089	0	0	0	0	0.062	0	0	0.47	0.482
IGF1	ITGAM	9606.ENSP00000302665	9606.ENSP00000441691	0	0	0	0	0.064	0	0	0.478	0.491
IGF1	INSR	9606.ENSP00000302665	9606.ENSP00000303830	0	0	0	0	0	0.945	0.8	0.978	0.999
IGF1	IGFBP3	9606.ENSP00000302665	9606.ENSP00000370473	0	0	0	0	0	0.871	0.8	0.991	0.999
IGF1R	IGFBP5	9606.ENSP00000268035	9606.ENSP00000233813	0	0	0	0	0.065	0	0	0.677	0.685
IGF1R	IGFBP4	9606.ENSP00000268035	9606.ENSP00000269593	0	0	0	0	0	0	0	0.637	0.637
IGF1R	IGFBP1	9606.ENSP00000268035	9606.ENSP00000275525	0	0	0	0	0	0	0	0.833	0.833
IGF1R	IGFBP3	9606.ENSP00000268035	9606.ENSP00000370473	0	0	0	0	0	0.27	0	0.803	0.85
IGF1R	INSR	9606.ENSP00000268035	9606.ENSP00000303830	0	0	0	0.957	0.062	0.854	0.8	0.983	0.971
IGF1R	IGF1	9606.ENSP00000268035	9606.ENSP00000302665	0	0	0	0	0	0.973	0.9	0.992	0.999
IGFBP1	IGF1R	9606.ENSP00000275525	9606.ENSP00000268035	0	0	0	0	0	0	0	0.833	0.833
IGFBP1	IGFBP4	9606.ENSP00000275525	9606.ENSP00000269593	0	0	0	0.813	0.098	0.927	0	0.854	0.942
IGFBP1	INSR	9606.ENSP00000275525	9606.ENSP00000303830	0	0	0	0	0	0	0	0.47	0.47
IGFBP1	IGF1	9606.ENSP00000275525	9606.ENSP00000302665	0	0	0	0	0.062	0.959	0.6	0.991	0.999
IGFBP3	IGFBP5	9606.ENSP00000370473	9606.ENSP00000233813	0	0	0	0.909	0.19	0	0.72	0.955	0.784
IGFBP3	IGF1R	9606.ENSP00000370473	9606.ENSP00000268035	0	0	0	0	0	0.27	0	0.803	0.85
IGFBP3	IGF1	9606.ENSP00000370473	9606.ENSP00000302665	0	0	0	0	0	0.871	0.8	0.991	0.999
IGFBP3	INSR	9606.ENSP00000370473	9606.ENSP00000303830	0	0	0	0	0	0	0	0.458	0.458
IGFBP4	IGF1R	9606.ENSP00000269593	9606.ENSP00000268035	0	0	0	0	0	0	0	0.637	0.637
IGFBP4	IGFBP1	9606.ENSP00000269593	9606.ENSP00000275525	0	0	0	0.813	0.098	0.927	0	0.854	0.942
IGFBP4	IGF1	9606.ENSP00000269593	9606.ENSP00000302665	0	0	0	0	0.085	0.973	0.6	0.989	0.999
IGFBP5	IGF1R	9606.ENSP00000233813	9606.ENSP00000268035	0	0	0	0	0.065	0	0	0.677	0.685
IGFBP5	IGFBP3	9606.ENSP00000233813	9606.ENSP00000370473	0	0	0	0.909	0.19	0	0.72	0.955	0.784
IGFBP5	IGF1	9606.ENSP00000233813	9606.ENSP00000302665	0	0	0	0	0	0.945	0.72	0.99	0.999
INSR	IGF1R	9606.ENSP00000303830	9606.ENSP00000268035	0	0	0	0.957	0.062	0.854	0.8	0.983	0.971
INSR	IGFBP1	9606.ENSP00000303830	9606.ENSP00000275525	0	0	0	0	0	0	0	0.47	0.47

INSR	IGF1	9606.ENSP00000303830	9606.ENSP00000302665	0	0	0	0	0	0.945	0.8	0.978	0.999
INSR	IGFBP3	9606.ENSP00000303830	9606.ENSP00000370473	0	0	0	0	0	0	0	0.458	0.458
ITGAL	RAC2	9606.ENSP00000349252	9606.ENSP00000249071	0	0	0	0	0.292	0	0	0.248	0.445
ITGAL	ICAM1	9606.ENSP00000349252	9606.ENSP00000264832	0	0	0	0	0.091	0.972	0.9	0.989	0.999
ITGAL	TLR4	9606.ENSP00000349252	9606.ENSP00000363089	0	0	0	0	0.069	0.059	0	0.39	0.418
ITGAL	CYBB	9606.ENSP00000349252	9606.ENSP00000367851	0	0	0	0	0.458	0	0	0.17	0.531
ITGAL	CXCR3	9606.ENSP00000349252	9606.ENSP00000362795	0	0	0	0	0.209	0	0	0.556	0.633
ITGAL	ITGAM	9606.ENSP00000349252	9606.ENSP00000441691	0	0	0	0.813	0.235	0	0.8	0.934	0.868
ITGAL	ITGB2	9606.ENSP00000349252	9606.ENSP00000380948	0	0	0	0	0.418	0.87	0.9	0.989	0.999
ITGAM	MSR1	9606.ENSP00000441691	9606.ENSP00000262101	0	0	0	0	0.128	0	0	0.528	0.571
ITGAM	ICAM1	9606.ENSP00000441691	9606.ENSP00000264832	0	0	0	0	0.096	0.462	0.9	0.989	0.999
ITGAM	LY96	9606.ENSP00000441691	9606.ENSP00000284818	0	0	0	0	0.127	0	0	0.459	0.507
ITGAM	NCF1	9606.ENSP00000441691	9606.ENSP00000289473	0	0	0	0	0.296	0	0	0.456	0.601
ITGAM	IGF1	9606.ENSP00000441691	9606.ENSP00000302665	0	0	0	0	0.064	0	0	0.478	0.491
ITGAM	CXCL10	9606.ENSP00000441691	9606.ENSP00000305651	0	0	0	0	0.126	0	0	0.672	0.701
ITGAM	HMGB1	9606.ENSP00000441691	9606.ENSP00000345347	0	0	0	0	0	0	0.9	0.641	0.962
ITGAM	ITGAL	9606.ENSP00000441691	9606.ENSP00000349252	0	0	0	0.813	0.235	0	0.8	0.934	0.868
ITGAM	NCF2	9606.ENSP00000441691	9606.ENSP00000356505	0	0	0	0	0.533	0	0	0.339	0.679
ITGAM	S100A8	9606.ENSP00000441691	9606.ENSP00000357722	0	0	0	0	0.332	0	0	0.207	0.447
ITGAM	S100A9	9606.ENSP00000441691	9606.ENSP00000357727	0	0	0	0	0.333	0	0	0.406	0.586
ITGAM	CXCR3	9606.ENSP00000441691	9606.ENSP00000362795	0	0	0	0	0.148	0	0	0.671	0.708
ITGAM	TLR4	9606.ENSP00000441691	9606.ENSP00000363089	0	0	0	0	0.17	0.059	0	0.832	0.858
ITGAM	CYBB	9606.ENSP00000441691	9606.ENSP00000367851	0	0	0	0	0.56	0	0	0.587	0.811
ITGAM	ITGB2	9606.ENSP00000441691	9606.ENSP00000380948	0	0	0	0	0.623	0.573	0.9	0.982	0.999
ITGAM	MX1	9606.ENSP00000441691	9606.ENSP00000381601	0	0	0	0	0.088	0	0	0.473	0.498
ITGB2	RAC2	9606.ENSP00000380948	9606.ENSP00000249071	0	0	0	0	0.643	0.058	0	0.631	0.865
ITGB2	ICAM1	9606.ENSP00000380948	9606.ENSP00000264832	0	0	0	0	0.113	0.835	0.9	0.989	0.999

ITGB2	NCF1	9606.ENSP0 0000380948	9606.ENSP0 0000289473	0	0	0	0	0.325	0.161	0	0.288	0.561
ITGB2	SKAP1	9606.ENSP0 0000380948	9606.ENSP0 0000338171	0	0	0	0	0.107	0	0	0.427	0.467
ITGB2	HMGB1	9606.ENSP0 0000380948	9606.ENSP0 0000345347	0	0	0	0	0.065	0	0.9	0.445	0.943
ITGB2	ITGAL	9606.ENSP0 0000380948	9606.ENSP0 0000349252	0	0	0	0	0.418	0.87	0.9	0.989	0.999
ITGB2	NCF2	9606.ENSP0 0000380948	9606.ENSP0 0000356505	0	0	0	0	0.653	0	0	0.346	0.763
ITGB2	S100A8	9606.ENSP0 0000380948	9606.ENSP0 0000357722	0	0	0	0	0.347	0	0	0.411	0.599
ITGB2	S100A9	9606.ENSP0 0000380948	9606.ENSP0 0000357727	0	0	0	0	0.533	0	0	0.157	0.59
ITGB2	CXCR3	9606.ENSP0 0000380948	9606.ENSP0 0000362795	0	0	0	0	0.213	0	0	0.323	0.444
ITGB2	TLR4	9606.ENSP0 0000380948	9606.ENSP0 0000363089	0	0	0	0	0.557	0.07	0	0.404	0.733
ITGB2	CYBB	9606.ENSP0 0000380948	9606.ENSP0 0000367851	0	0	0	0	0.568	0	0	0.323	0.695
ITGB2	ITGAM	9606.ENSP0 0000380948	9606.ENSP0 0000441691	0	0	0	0	0.623	0.573	0.9	0.982	0.999
LMNB1	EMD	9606.ENSP0 0000261366	9606.ENSP0 0000358857	0	0	0	0	0.084	0.317	0.9	0.988	0.999
LMNB1	BANF1	9606.ENSP0 0000261366	9606.ENSP0 0000310275	0	0	0	0	0.063	0.378	0.9	0.986	0.999
LY96	ICAM1	9606.ENSP0 0000284818	9606.ENSP0 0000264832	0	0	0	0	0.135	0	0	0.391	0.45
LY96	CXCL10	9606.ENSP0 0000284818	9606.ENSP0 0000305651	0	0	0	0	0.11	0	0	0.459	0.497
LY96	ITGAM	9606.ENSP0 0000284818	9606.ENSP0 0000441691	0	0	0	0	0.127	0	0	0.459	0.507
LY96	S100A8	9606.ENSP0 0000284818	9606.ENSP0 0000357722	0	0	0	0	0.117	0	0.9	0.5	0.951
LY96	S100A9	9606.ENSP0 0000284818	9606.ENSP0 0000357727	0	0	0	0	0.118	0	0.9	0.609	0.962
LY96	HMGB1	9606.ENSP0 0000284818	9606.ENSP0 0000345347	0	0	0	0	0	0.417	0.9	0.978	0.998
LY96	TLR4	9606.ENSP0 0000284818	9606.ENSP0 0000363089	0	0	0	0	0.176	0.982	0.9	0.994	0.999
ME1	SCD	9606.ENSP0 0000358719	9606.ENSP0 0000359380	0	0	0	0	0.112	0	0	0.444	0.485
MSR1	ITGAM	9606.ENSP0 0000262101	9606.ENSP0 0000441691	0	0	0	0	0.128	0	0	0.528	0.571
MSR1	TLR4	9606.ENSP0 0000262101	9606.ENSP0 0000363089	0	0	0	0	0.118	0	0	0.639	0.668
MX1	OASL	9606.ENSP0 0000381601	9606.ENSP0 0000257570	0	0	0	0	0.962	0.149	0.9	0.794	0.999
MX1	CXCL10	9606.ENSP0 0000381601	9606.ENSP0 0000305651	0	0	0	0	0.416	0	0	0.703	0.819
MX1	CXCR3	9606.ENSP0 0000381601	9606.ENSP0 0000362795	0	0	0	0	0.08	0.056	0	0.395	0.429
MX1	TLR4	9606.ENSP0 0000381601	9606.ENSP0 0000363089	0	0	0	0	0.062	0.076	0	0.464	0.495

MX1	ITGAM	9606.ENSP00000381601	9606.ENSP00000441691	0	0	0	0	0.088	0	0	0.473	0.498
MX1	IFI27	9606.ENSP00000381601	9606.ENSP00000483430	0	0	0	0	0.657	0	0.9	0.687	0.988
NCF1	RAC2	9606.ENSP00000289473	9606.ENSP00000249071	0	0	0	0	0.156	0.32	0.8	0.963	0.995
NCF1	CYBA	9606.ENSP00000289473	9606.ENSP00000261623	0	0	0	0	0.155	0.748	0.9	0.993	0.999
NCF1	ICAM1	9606.ENSP00000289473	9606.ENSP00000264832	0	0	0	0	0.069	0	0	0.541	0.555
NCF1	TLR4	9606.ENSP00000289473	9606.ENSP00000363089	0	0	0	0	0.108	0	0	0.511	0.545
NCF1	ITGB2	9606.ENSP00000289473	9606.ENSP00000380948	0	0	0	0	0.325	0.161	0	0.288	0.561
NCF1	ITGAM	9606.ENSP00000289473	9606.ENSP00000441691	0	0	0	0	0.296	0	0	0.456	0.601
NCF1	S100A8	9606.ENSP00000289473	9606.ENSP00000357722	0	0	0	0	0.23	0	0.6	0.09	0.695
NCF1	S100A9	9606.ENSP00000289473	9606.ENSP00000357727	0	0	0	0	0.23	0	0.6	0.14	0.712
NCF1	RAC1	9606.ENSP00000289473	9606.ENSP00000348461	0	0	0	0	0.062	0.441	0.9	0.48	0.969
NCF1	NCF2	9606.ENSP00000289473	9606.ENSP00000356505	0	0	0	0	0.305	0.979	0.9	0.996	0.999
NCF1	CYBB	9606.ENSP00000289473	9606.ENSP00000367851	0	0	0	0	0.303	0.213	0.9	0.992	0.999
NCF2	RAC2	9606.ENSP00000356505	9606.ENSP00000249071	0	0	0	0	0.311	0.735	0.8	0.989	0.999
NCF2	CYBA	9606.ENSP00000356505	9606.ENSP00000261623	0	0	0	0	0.156	0.462	0.9	0.993	0.999
NCF2	NCF1	9606.ENSP00000356505	9606.ENSP00000289473	0	0	0	0	0.305	0.979	0.9	0.996	0.999
NCF2	RAC1	9606.ENSP00000356505	9606.ENSP00000348461	0	0	0	0	0.065	0.873	0.9	0.933	0.999
NCF2	TLR4	9606.ENSP00000356505	9606.ENSP00000363089	0	0	0	0	0.26	0	0	0.492	0.608
NCF2	ITGAM	9606.ENSP00000356505	9606.ENSP00000441691	0	0	0	0	0.533	0	0	0.339	0.679
NCF2	ITGB2	9606.ENSP00000356505	9606.ENSP00000380948	0	0	0	0	0.653	0	0	0.346	0.763
NCF2	S100A9	9606.ENSP00000356505	9606.ENSP00000357727	0	0	0	0	0.564	0.27	0.6	0.145	0.876
NCF2	S100A8	9606.ENSP00000356505	9606.ENSP00000357722	0	0	0	0	0.563	0.27	0.6	0.451	0.92
NCF2	CYBB	9606.ENSP00000356505	9606.ENSP00000367851	0	0	0	0	0.382	0.462	0.9	0.992	0.999
OASL	CXCL10	9606.ENSP00000257570	9606.ENSP00000305651	0	0	0	0	0.639	0	0	0.522	0.82
OASL	IFI27	9606.ENSP00000257570	9606.ENSP00000483430	0	0	0	0	0.117	0	0.9	0.67	0.968
OASL	MX1	9606.ENSP00000257570	9606.ENSP00000381601	0	0	0	0	0.962	0.149	0.9	0.794	0.999
QPCT	SOAT1	9606.ENSP00000344829	9606.ENSP00000356591	0	0	0	0	0.062	0	0	0.41	0.422

RAC1	RAC2	9606.ENSP00000348461	9606.ENSP00000249071	0	0	0.447	0.983	0.089	0	0.5	0.602	0.533
RAC1	CYBA	9606.ENSP00000348461	9606.ENSP00000261623	0	0	0	0	0.064	0.213	0.9	0.409	0.95
RAC1	NCF1	9606.ENSP00000348461	9606.ENSP00000289473	0	0	0	0	0.062	0.441	0.9	0.48	0.969
RAC1	CYBB	9606.ENSP00000348461	9606.ENSP00000367851	0	0	0	0	0.093	0.242	0.9	0.62	0.97
RAC1	NCF2	9606.ENSP00000348461	9606.ENSP00000356505	0	0	0	0	0.065	0.873	0.9	0.933	0.999
RAC2	TLR4	9606.ENSP00000249071	9606.ENSP00000363089	0	0	0	0	0.133	0.125	0	0.298	0.421
RAC2	ITGAL	9606.ENSP00000249071	9606.ENSP00000349252	0	0	0	0	0.292	0	0	0.248	0.445
RAC2	RAC1	9606.ENSP00000249071	9606.ENSP00000348461	0	0	0.447	0.983	0.089	0	0.5	0.602	0.533
RAC2	ICAM1	9606.ENSP00000249071	9606.ENSP00000264832	0	0	0	0	0.125	0	0	0.549	0.589
RAC2	S100A9	9606.ENSP00000249071	9606.ENSP00000357727	0	0	0	0	0.139	0	0.6	0	0.64
RAC2	S100A8	9606.ENSP00000249071	9606.ENSP00000357722	0	0	0	0	0.117	0	0.6	0.09	0.65
RAC2	ITGB2	9606.ENSP00000249071	9606.ENSP00000380948	0	0	0	0	0.643	0.058	0	0.631	0.865
RAC2	CYBA	9606.ENSP00000249071	9606.ENSP00000261623	0	0	0	0	0.227	0	0.8	0.959	0.993
RAC2	NCF1	9606.ENSP00000249071	9606.ENSP00000289473	0	0	0	0	0.156	0.32	0.8	0.963	0.995
RAC2	CYBB	9606.ENSP00000249071	9606.ENSP00000367851	0	0	0	0	0.259	0.237	0.8	0.988	0.998
RAC2	NCF2	9606.ENSP00000249071	9606.ENSP00000356505	0	0	0	0	0.311	0.735	0.8	0.989	0.999
S100A8	RAC2	9606.ENSP00000357722	9606.ENSP00000249071	0	0	0	0	0.117	0	0.6	0.09	0.65
S100A8	CYBA	9606.ENSP00000357722	9606.ENSP00000261623	0	0	0	0	0.087	0	0.6	0.042	0.619
S100A8	LY96	9606.ENSP00000357722	9606.ENSP00000284818	0	0	0	0	0.117	0	0.9	0.5	0.951
S100A8	NCF1	9606.ENSP00000357722	9606.ENSP00000289473	0	0	0	0	0.23	0	0.6	0.09	0.695
S100A8	NCF2	9606.ENSP00000357722	9606.ENSP00000356505	0	0	0	0	0.563	0.27	0.6	0.451	0.92
S100A8	ITGAM	9606.ENSP00000357722	9606.ENSP00000441691	0	0	0	0	0.332	0	0	0.207	0.447
S100A8	ITGB2	9606.ENSP00000357722	9606.ENSP00000380948	0	0	0	0	0.347	0	0	0.411	0.599
S100A8	CYBB	9606.ENSP00000357722	9606.ENSP00000367851	0	0	0	0	0.347	0	0.6	0.15	0.758
S100A8	TLR4	9606.ENSP00000357722	9606.ENSP00000363089	0	0	0	0	0.152	0	0.9	0.703	0.972
S100A8	S100A9	9606.ENSP00000357722	9606.ENSP00000357727	0	0	0	0.801	0.995	0.95	0.9	0.987	0.999
S100A9	RAC2	9606.ENSP00000357727	9606.ENSP00000249071	0	0	0	0	0.139	0	0.6	0	0.64

S100A9	CYBA	9606.ENSP00000357727	9606.ENSP00000261623	0	0	0	0	0.087	0	0.6	0.072	0.632
S100A9	LY96	9606.ENSP00000357727	9606.ENSP00000284818	0	0	0	0	0.118	0	0.9	0.609	0.962
S100A9	NCF1	9606.ENSP00000357727	9606.ENSP00000289473	0	0	0	0	0.23	0	0.6	0.14	0.712
S100A9	NCF2	9606.ENSP00000357727	9606.ENSP00000356505	0	0	0	0	0.564	0.27	0.6	0.145	0.876
S100A9	S100A8	9606.ENSP00000357727	9606.ENSP00000357722	0	0	0	0.801	0.995	0.95	0.9	0.987	0.999
S100A9	ITGAM	9606.ENSP00000357727	9606.ENSP00000441691	0	0	0	0	0.333	0	0	0.406	0.586
S100A9	ITGB2	9606.ENSP00000357727	9606.ENSP00000380948	0	0	0	0	0.533	0	0	0.157	0.59
S100A9	CYBB	9606.ENSP00000357727	9606.ENSP00000367851	0	0	0	0	0.351	0	0.6	0.154	0.761
S100A9	TLR4	9606.ENSP00000357727	9606.ENSP00000363089	0	0	0	0	0.183	0	0.9	0.883	0.989
SCD	FABP5	9606.ENSP00000359380	9606.ENSP00000297258	0	0	0	0	0.062	0	0	0.488	0.499
SCD	SOAT1	9606.ENSP00000359380	9606.ENSP00000356591	0	0	0	0	0.108	0	0	0.437	0.476
SCD	ME1	9606.ENSP00000359380	9606.ENSP00000358719	0	0	0	0	0.112	0	0	0.444	0.485
SKAP1	ITGB2	9606.ENSP00000338171	9606.ENSP00000380948	0	0	0	0	0.107	0	0	0.427	0.467
SOAT1	QPCT	9606.ENSP00000356591	9606.ENSP00000344829	0	0	0	0	0.062	0	0	0.41	0.422
SOAT1	SCD	9606.ENSP00000356591	9606.ENSP00000359380	0	0	0	0	0.108	0	0	0.437	0.476
SRPX	CYBB	9606.ENSP00000367794	9606.ENSP00000367851	0	0	0	0	0	0	0	0.536	0.536
TLR4	RAC2	9606.ENSP00000363089	9606.ENSP00000249071	0	0	0	0	0.133	0.125	0	0.298	0.421
TLR4	CYBA	9606.ENSP00000363089	9606.ENSP00000261623	0	0	0	0	0.062	0	0	0.392	0.405
TLR4	MSR1	9606.ENSP00000363089	9606.ENSP00000262101	0	0	0	0	0.118	0	0	0.639	0.668
TLR4	ICAM1	9606.ENSP00000363089	9606.ENSP00000264832	0	0	0	0	0.083	0	0	0.786	0.796
TLR4	LY96	9606.ENSP00000363089	9606.ENSP00000284818	0	0	0	0	0.176	0.982	0.9	0.994	0.999
TLR4	NCF1	9606.ENSP00000363089	9606.ENSP00000289473	0	0	0	0	0.108	0	0	0.511	0.545
TLR4	IGF1	9606.ENSP00000363089	9606.ENSP00000302665	0	0	0	0	0.062	0	0	0.47	0.482
TLR4	CXCL10	9606.ENSP00000363089	9606.ENSP00000305651	0	0	0	0	0.078	0	0	0.959	0.961
TLR4	HMGB1	9606.ENSP00000363089	9606.ENSP00000345347	0	0	0	0	0	0.632	0.9	0.989	0.999
TLR4	ITGAL	9606.ENSP00000363089	9606.ENSP00000349252	0	0	0	0	0.069	0.059	0	0.39	0.418
TLR4	NCF2	9606.ENSP00000363089	9606.ENSP00000356505	0	0	0	0	0.26	0	0	0.492	0.608

TLR4	S100A8	9606.ENSP0 0000363089	9606.ENSP0 0000357722	0	0	0	0	0.152	0	0.9	0.703	0.972
TLR4	S100A9	9606.ENSP0 0000363089	9606.ENSP0 0000357727	0	0	0	0	0.183	0	0.9	0.883	0.989
TLR4	CXCR3	9606.ENSP0 0000363089	9606.ENSP0 0000362795	0	0	0	0	0.062	0	0	0.469	0.48
TLR4	MX1	9606.ENSP0 0000363089	9606.ENSP0 0000381601	0	0	0	0	0.062	0.076	0	0.464	0.495
TLR4	ITGB2	9606.ENSP0 0000363089	9606.ENSP0 0000380948	0	0	0	0	0.557	0.07	0	0.404	0.733
TLR4	CYBB	9606.ENSP0 0000363089	9606.ENSP0 0000367851	0	0	0	0	0.296	0.059	0	0.692	0.778
TLR4	ITGAM	9606.ENSP0 0000363089	9606.ENSP0 0000441691	0	0	0	0	0.17	0.059	0	0.832	0.858
TSPAN8	ICAM1	9606.ENSP0 0000377003	9606.ENSP0 0000264832	0	0	0	0	0.052	0	0	0.472	0.479

PPI, protein–protein interaction; STRING, Search Tool for the Retrieval of Interacting Genes; MCODE, Minimal Common Oncology Data Elements.
